# Impact of Quiet Time on Psychological Outcomes of Neonatal Intensive Care Unit Nurses in Jeddah, Saudi Arabia: A Cross-Sectional Study

**DOI:** 10.7759/cureus.50307

**Published:** 2023-12-11

**Authors:** Ahmad Ismail, Ashwag Imam, Minerva Raguini, Dina Hassan, Aziza Ali, Abdulaziz Alkhotani

**Affiliations:** 1 Neonatal Intensive Care, Fakeeh College for Medical Sciences, Jeddah, SAU; 2 Pediatrics, Umm Al-Qura University, Makkah, SAU; 3 Pediatrics, Dr. Soliman Fakeeh Hospital, Jeddah, SAU

**Keywords:** depression, anxiety, stress, nurse, saudi arabia, neonatal intensive care unit (nicu), quiet-time

## Abstract

Background and objective: Although quiet time is implemented in neonatal intensive care units (NICUs) for the benefit of infants, it may also positively impact the psychological outcomes of healthcare professionals. Several studies have examined the impact of quiet-time implementation on patients; however, there is a paucity of research assessing its impact on the psychological outcomes of NICU nurses, particularly in Saudi Arabia.

Objective and methods: This study aimed to assess the impact of quiet time on the psychological outcomes (stress, anxiety, and depression) of NICU nurses in Jeddah, Saudi Arabia. A cross-sectional design was used for this study. A total of 87 NICU nurses from two hospitals participated in this study. One group did not practice quiet time, while the second group did. A questionnaire survey assessed participants’ demographic characteristics, and their depression, anxiety, and stress were assessed using the depression, anxiety, and stress scale-21 (DASS-21). The data were analyzed for frequency, percentage, mean, and standard deviation (SD). Bivariate analysis, independent t-tests, and one-way analysis of variance were used to test the differences between variables and groups. Pearson’s correlation coefficient (*r*) was used to analyze the relationships between continuous variables and perceived stress, anxiety, and depression.

Results: A substantial number of NICU nurses perceived stress, anxiety, and depression; however, there were no significant differences in perceived stress, anxiety, and depression between the nurses who worked in NICUs that applied quiet time and NICUs that did not (P ≤ 0.05).

Conclusion: This study found no statistically significant relationship between quiet-time implementation and perceived stress, anxiety, or depression among NICU nurses. Further research with a larger sample size or increased quiet-time implementation may be required.

## Introduction

Noise is prevalent and inevitable in the ICU [1-4]. When noise exceeds the recommended level, it can be harmful to patients as well as healthcare professionals [1,5,6]. At the patient level, increased noise levels in the ICUs may result in sleep deprivation, decreased healing, increased pain perception, delirium, increased length of stay, annoyance, increased stress level, and impaired well-being. At the healthcare professional level, increased noise may lead to increased medical errors, increased stress levels, and reduced performance [5,7-13]. Research indicates that sound levels exceed the recommended noise level in ICUs [1,6,14,15].

Healthcare professionals contribute 30% to 60% of hospital noise [7]. Operational noise can be attributed to staff talk and equipment (e.g., ventilators, intravenous infusion pumps, and cardiac monitors), while structural noise can be from ventilation, air conditioning, and automatic door opening and closing. Eighty percent of noise sources are from monitor or ventilator alarms and speech [1,16,17]. However, 90% of monitor alarms are false positives [18]. The nursing station is one of the loudest places in the hospital because staff communication, telephones, and central monitoring stations are typically located there [1,8]. According to a study, 62% of the noise in ICUs is completely or partially avoidable [19]. From the patient's perspective, the most anxious noise comes from healthcare professionals during shift change [20]. Another study has found that ICU staff is the major contributor to noise [21], suggesting that noise in the ICU can be reduced as it is linked to modifiable factors.

In the hospital, the environment should be kept quiet (the noise level should not exceed 45 dB during the day and 35 dB at night) [1]. In the NICU, the noise level exceeds the normal hospital noise level by two to three times [22]. Babies in the NICU undergo a critical period of neurodevelopment. Those babies suffer negative consequences from the high noise level [22,23]. Infants are less resistant to the harmful effects of noise [24]. A noisy NICU can increase infants’ energy expenditure, compromising their growth and development [25]. The harmful effects of high noise levels reach healthcare professionals, especially NICU nurses, who may stay inside the NICU for a whole shift daily. These harmful effects include physical and psychological consequences such as headaches, hypertension, burnout, low-performance levels, and negative psychological outcomes [3,22,26,27]. Therefore, implementing noise reduction solutions in the NICU may alleviate the negative consequences for babies and healthcare professionals [22].

Both patients and nurses benefit from daily quiet periods. Quiet time refers to reducing light and sound for a period of time [3,28]. During quiet time, measures should be implemented, including limiting conversations, eliminating environmental noise, and dimming lights [8]. Implementing quiet time protocols in the NICU results in positive outcomes for patients and healthcare professionals [29-31]. Quiet time contributes to healthier workplace environments [30,31]. Using quiet time results in a lower stress level among ICU nurses [31]. It is an energy-saving intervention in the workplace environment [31].

Several studies have examined the impact of quiet-time implementation in hospitals. Quiet time has many benefits for patients; however, few studies have investigated its benefits for nurses. Moreover, there is a lack of research in Saudi Arabia evaluating the impact of quiet time on NICU nurses’ psychological outcomes (stress, anxiety, and depression). Hence, this study aimed to assess the impact of quiet time on perceived stress, anxiety, and depression in NICU nurses in Jeddah, Saudi Arabia.

## Materials and methods

Research design

A cross-sectional design was used in this study. The independent variable in the study was the implementation of quiet time in the NICU, and the dependent variable was the psychological outcomes of NICU nurses (stress, anxiety, and depression). Data were collected for three months between January and April 2023 from NICU nurses at two hospitals in Jeddah, Saudi Arabia. First, we identified one NICU that applied the quiet time protocol as a daily practice and one that did not. Second, we collected data from the NICU nurses working in these two units. Quiet time was applied in one NICU according to unit flow and patient care activity. The quiet time is applied for one hour on the day shift from 1300 to 1400H or 1400 to 1500H and one hour on the night shift from 0100 to 0200H or 0200H to 0300H. During the quiet time, NICU nurses took all possible measures to keep the unit quiet for the patients as well as for the healthcare professionals. These measures include posting signs in a well-visible place to remind staff to keep quiet, with captions such as Don’t disturb, Quiet hours...shhh, and A quiet place is a healing environment. These signs were placed at the entrance of the NICU and inside the unit. In addition, the lights were dimmed, the staff lowered their voices and conversations, cellphones were muted, fast responses to any alarm were implemented, incubators were covered, and visitors were instructed to keep their voices low. The NICU nurses were responsible for controlling the implementation of the quiet time hours. 

Participants and sampling

The target population for this study comprised nurses working in the NICUs of Dr. Soliman Fakeeh Hospital (DSFH) and King Abdulaziz Hospital (KAAH) in Jeddah, Saudi Arabia. The number of NICU nurses employed at these two facilities is 90. Based on Slovin’s formula, the required sample should follow n = N / (1 + Ne^2). Therefore, the minimum required sample is 90/(1 + 90*0.05*0.05) = 73. This study recruited 87 nurses, which is sufficient to represent the population of the two hospitals. Nurses were selected for the study using convenience sampling. This type of selection, called non-probability or non-random sampling, involves selecting members of a target population who meet specific criteria. The researchers ensured that the study participants met the following criteria: nurses working in the NICU with at least three months of experience in the unit; male and female nurses with a diploma, bachelor’s, or master’s degree; willingness to participate in the study; and Saudi and non-Saudi nurses. Participants who did not meet the inclusion criteria were excluded.

The setting of the study

Both hospitals included in the study are located in Jeddah, Saudi Arabia, and are renowned for their internationally accredited and quality-recognized critical care units. The KAAH is a 436-bed government hospital. Established in June 1990, it offers inpatient and outpatient services to men and women of all ages. The DSFH is a prominent healthcare facility in the region, which currently cares for over 700,000 patients annually and has 475 beds, 120 clinics, and 15 operating theaters. In its quest to provide quality medical services across all platforms of patient needs, DSFH has embarked on projects that will see its capacity expand significantly. When completed, it is expected to have 810 beds along with 393 clinics and 39 operating theaters. The present study was conducted at the NICUs of both KAAH (20 NICU beds) and DSFH (30 NICU beds).

Research instruments

Since English is the official language of healthcare professionals in the participating hospitals, all assessment tools were in English. The questionnaire comprises three main sections. Section 1 dealt with 10 demographic items, including age, gender, marital status, citizenship, education level, years of experience in the NICU, position, work shift, salary, and hospital type. Section 2 contains three closed-ended questions and one open-ended question related to quiet time. Section 3 deploys the depression, anxiety, and stress scale-21 (DASS-21), which comprises three self-report scales to assess perceived depression, anxiety, and stress [32].

In DASS-21, depression is measured using seven items on a scale of 0 to 3 (0 = did not apply to me at all, and 3 = applied to me very much or most of the time). As the DASS-21 is the short version of the DASS-42, the sum of each individual score must be multiplied by two. Scores ranging from 0 to 9 indicate normal, while scores ranging from 10 to 13, 14 to 20, 21 to 27, and ≥ 28 indicate mild, moderate, severe, and extremely severe perceptions of depression, respectively. Stress was measured using seven items rated from 0 to 3, similar to the depression scale. Scores from 0 to 14, 15 to 18, 19 to 25, 26 to 33, and ≥ 34 indicate normal, mild, moderate, severe, and extremely severe perceptions of stress, respectively. Anxiety was measured using seven items rated from 0 to 3, similar to the depression and stress scales. Scores of 0 to 7, 8 to 9, 10 to 14, 15 to 19, and ≥ 20 indicate normal, mild, moderate, severe, and extremely severe perceptions of anxiety. The validity and reliability of the DASS-21 are well established by previous research [33-35]. Cronbach’s alpha for the three subscales has been found to be acceptable, at more than 0.70, with excellent internal consistency as well as discriminative, concurrent, and convergent validity [33]. The scale is open-access, and no permission is required to use it [36].

Procedure

Following ethical approval, permission was sought from the Chief Nursing Officers at both hospitals was sought to access the nurses in their NICUs. The data collection period commenced in January 2023 and continued until April 2023. Data were collected only when all the necessary approvals had been obtained. After the Chief Nursing Officer's consent, potential participants were invited to participate in the study. Participants were given a questionnaire through the SurveyMonkey (San Mateo, CA, USA) link and a quick response (QR) code that outlined the purpose of this research and provided them with knowledge regarding the possible benefits or risks that may arise during the investigation.

Data analysis

The SPSS Statistics version 26.0 (IBM Corp., Armonk, NY, USA) was used to analyze the data. The data were screened to ensure integrity. Missing data were analyzed and treated using the multiple imputation method. The data were analyzed for frequency, percentage, mean, and standard deviation (SD). A bivariate analysis was conducted between the study variables and perceived stress, anxiety, and depression. An independent t-test was used to assess the differences between the two independent groups in terms of perceived stress, anxiety, and depression. One-way analysis of variance (ANOVA) was used to test the differences between three or more groups in terms of perceived stress, anxiety, and depression. Pearson’s correlation coefficient (r) was used to analyze the relationships between continuous variables and perceived stress, anxiety, and depression.

Ethical consideration

Ethical approval was obtained from the Research Ethics Committee of Fakeeh College for Medical Sciences (approval no. 377/IRB/2022) and the Ministry of Health in Jeddah (A01577). The study plans were supported by research registration, publication, and dissemination. The first section of the survey asked participants to provide their consent to complete the survey. The remaining questions would appear only if participants agreed to participate. Participation in and completion of the survey implies consent to participate.

Participants were informed that their participation was voluntary and anonymous; they could withdraw at any time before the survey was submitted; their data were encrypted, and their personal information would be kept confidential and protected by a password; access would remain only with the researchers; their data would be presented and published in the aggregate; and a secure deletion process would destroy the data after five years. Participants were not required to provide identifiers. Neither were any electronic identifiers, such as IP addresses, tracked. However, SurveyMonkey prohibited the use of more than one response per device and ensured data protection.

## Results

Eighty-seven participants out of 90 completed the survey. All participants (100%) were women with a mean age of 34 years and an average of eight years of experience in the NICU. Most were staff nurses (85%), had a bachelor's degree in nursing (84%), were non-Saudis (62%), and worked in public hospitals (58%). Nearly half of the participants were married (52%), received a monthly salary between 5001 and 10000 Saudi riyals, and worked day shifts in the previous month (Table 1).

**Table 1:** Demographic characteristics of the participants

Characteristics	Frequency	Percentage	Mean	SD
Age			34	8
Years of experience in NICU			8	6
Gender				
Women	87	100%		
Marital status				
Single	42	48%		
Married	45	52%		
Citizenship				
Saudi	33	38%		
Non-Saudi	54	62%		
Education				
College diploma	12	14%		
Bachelor’s degree	73	84%		
Master's and above	2	2%		
Hospital type				
Public	50	57%		
Private	37	43%		
Position				
Staff nurse	74	85.1%		
Charge nurse	8	9.2%		
Nurse manager	2	2.3%		
Nurse educator	2	2.3%		
Clinical resource nurse	1	1.1%		
Common work shifts last month				
Night	41	47%		
Day	46	53%		

Quiet time was implemented as a protocol in one of the two hospitals that participated in this study. It was implemented in the NICU of the private hospital (43% of participants). The duration of quiet time was one hour (57%), and it was implemented between 1 pm and 3 pm (54%) and between 1 am and 3 am (46%) (Table 2).

**Table 2:** Quiet time implementation in the NICU

Variables	Frequency	Percentage
Quiet time		
No	50	57%
Yes	37	43%
Quiet time duration		
Public/no quiet time	50	57%
1 hour	37	43%
Quiet time implementation period		
1 am to 3 am	17	46%
1 pm to 3 pm	20	54%

The mean perceived stress score was 13 (SD = 9). Most participants perceived no stress (63%), 12% reported mild stress, 17% expressed moderate stress, and 8% reported severe to extremely severe stress (Table 3).

**Table 3:** Perceived stress of NICU nurses as per DASS-21 stress subscale DASS-21: Depression, anxiety, and stress scale-21

Stress level	Score	Frequency	Percentage
Normal	0-14	55	63
Mild	15-18	10	12
Moderate	19-25	15	17
Severe	26-33	5	6
Extremely severe	≥ 34	2	2

The mean perceived anxiety score was 11 (SD = 8). Half of the participants perceived moderate to extremely severe anxiety (50%), 37% expressed no anxiety, and 13% reported mild anxiety (Table 4).

**Table 4:** Perceived anxiety of NICU nurses as per DASS-21 anxiety subscale DASS-21: Depression, anxiety, and stress scale-21

Anxiety level	Score	Frequency	Percentage
Normal	0-7	32	36.8
Mild	8-9	11	12.6
Moderate	10-14	21	24.1
Severe	15-19	11	12.6
Extremely severe	≥ 20	12	13.6

The mean perceived depression score was 10 (SD = 9). Forty-six percent of the participants perceived some degree of depression; 17% perceived mild depression, 15% expressed moderate depression, 9% reported severe depression, and 5% stated extremely severe depression (Table 5).

**Table 5:** Perceived depression of NICU nurses as per DASS-21 depression subscale DASS-21: Depression, anxiety, and stress scale-21

Depression level	Score	Frequency	Percentage
Normal	0-9	47	54
Mild	10-13	15	17
Moderate	14-20	13	15
Severe	21-27	8	9
Extremely severe	≥ 28	4	5

There was no statistically significant relationship between the majority of the study variables and perceived stress, anxiety, and depression among NICU nurses (p≥0.05). There was no significant difference in perceived stress, anxiety, or depression between nurses who worked in NICUs that applied quiet time and those who did not (Table 6).

**Table 6:** Relationship between study variables and perceived stress, anxiety, and depression t: Independent t-test; F: F test (one-way ANOVA); r: Pearson correlation coefficient

Variables	Perceived stress			Perceived anxiety			Perceived depression		
	Mean	Test	P	Mean	Test	P	Mean	Test	P
Marital status									
Single	13	t= 0.2	0.8	13	t= 1	0.3	10	t= 0.04	0.9
Married	13			11			10		
Citizenship									
Saudi	13	t= 0.17	0.8	12	t= 0.7	0.5	11	t= 1.1	0.3
Non-Saudi	13			11			9		
Education									
College diploma	12	F= 0.1	0.7	11	F= 0.1	0.8	9	F= 0.1	0.7
Bachelor's	13			12			10		
≥ Master's	13			11			10		
Hospital type									
Public	13	t= 0.6	0.5	11	t= 1	0.3	10	t= 0.08	0.9
Private	14			13			10		
Position									
Staff nurse	14	F= 0.8	0.5	12	F= 0.9	0.4	10	F= 0.4	0.8
Charge nurse	9			9			8		
Nurse manager	6			2			4		
Nurse educator	14			14			14		
Clinical resource nurse	20			18			6		
Monthly salary									
0-5000 SR	11	F= 1	0.3	11	F= 0.8	0.5	8	F= 1	0.3
5001-10000 SR	13			12			11		
10001-15000 SR	15			13			12		
15001-20000 SR	3			3			1		
≥20000 SR	14			8			8		
Previous month's work shifts									
Night	12	t= 0.7	0.5	10	t= 1.5	0.1	10	t= 0.13	0.9
Day	14			13			10		
Quiet time									
No	13	t= 0.6	0.5	11	t= 0.9	0.3	10	t= 0.1	0.9
Yes	14			13			10		
	Perceived stress			Perceived anxiety			Perceived depression		
Continuous variables	Mean	Test	P	Mean	Test	P	Mean	Test	P
Experience in NICU		r= -0.1	0.4		r= -0.21	0.07		r= -0.15	0.2
Age		r= -0.15	0.18		r= -0.19	0.11		r=-0.18	0.12

For the open-ended questions, participants reported that quiet time benefited babies in the NICU (10%). Quiet time was also a power booster for the staff to regain comfort (12%) (Table 7).

**Table 7:** Responses of NICU nurses to open-ended questions on their perception of quiet time

Item	Frequency	%
Quiet time makes babies sleep well, quietly, and more comfortably	9	10
Quiet time is a power booster at least for a few hours, for the staff to regain if they get tired	10	12
Quiet time can be more effective in the NICU if visiting hours are more restricted	2	2

## Discussion

Nearly half of the NICU nurses in this study perceived negative psychological outcomes (37% mild to extremely severe stress, 63% mild to extremely severe anxiety, and 46% mild to extremely severe depression). There were no statistically significant relationships between most of the study variables and perceived stress, anxiety, or depression among NICU nurses. Most of the quiet time was one hour, implemented in the early morning and afternoon in NICUs. There was no statistically significant difference in perceived stress, anxiety, or depression between nurses working in NICUs who applied quiet time and those who did not.

Since this study found no significant relationship between quiet time and perceived stress, anxiety, or depression among NICU nurses from the two participating hospitals in Jeddah, Saudi Arabia, it can be said that the implementation of quiet time in the NICU did not reduce stress, anxiety, or depression among nurses. Despite this, participants who answered the open-ended questions reported that quiet time was a power booster that helped them regain their comfort to continue working, and it also helped babies sleep more quietly and comfortably in the NICUs. This will eventually reflect positively on the nurses’ psychological status, as pediatric nurses are affected by the conditions of their patients. In addition, the period of quiet time, as reported by the participants of this study, was limited to one hour, which may not be enough to produce a reduction in the stress, anxiety, and depression of the NICU nurses. One study found a significant reduction in noise levels after implementing quiet time for two hours every 12 hours from 3 a.m. to 5 a.m. and 3 p.m. to 5 p.m. in the ICU [8].

Moreover, previous research has shown that quiet time in hospitals, particularly in ICUs, provides many benefits, particularly in NICUs, where it is associated with more silence, fewer electronic sounds, and less talk than non-quiet time. Implementing quiet time decreases noise and exposure to electronic sounds in the NICU without sacrificing patient care [29,37,38]. Applebaum et al. [39] found that most participants reported lower noise levels after quiet-time interventions. Sixty percent of the participants reported that one hour of quiet time helped create a quieter, more restful atmosphere throughout the day. Quiet time had a positive effect on the patients and their relatives and showed an improved patient experience [40]. Quiet-time interventions can improve patients' sleep and satisfaction and have positive physiological effects such as improved restfulness and reduced respiratory rates, sedative administration, and delirium incidence [30,31]. Quiet time encourages a positive workplace atmosphere, particularly for nurses [30].

The majority of participants in this study were ICU nurses, who were more likely to perceive stress, anxiety, and depression than other nurses. In a study conducted by McGough et al. [41], uncontrolled noise in the hospital setting can have a negative physiological and psychological impact on patients and nurses. Increased noise levels in the ICU lead to several negative outcomes, including stress, anxiety, depression, sleep deprivation, decreased healing, increased pain perception, and delirium [5]. It also induces annoyance, heightened stress levels, impaired well-being, and reduced performance [7,8,10].

Our findings, despite their negative outcomes, provide recommendations for future studies. Intervention programs are needed to identify the causes of negative psychological outcomes, and measures to decrease the percentage of such negative outcomes are imperative. Multivariate research is needed to identify relevant factors affecting psychological outcomes among nurses in Saudi Arabia. The models for such research should include the participants' family, community, and physical and psychological health factors. A larger sample of NICU nurses should be investigated in other cities in Saudi Arabia to obtain comparable results. Future research is also needed on strategies for reducing stress, depression, and anxiety among NICU nurses, as this study established the effect of quiet time on psychological outcomes among NICU nurses. Since self-reports may be inherently biased, future research may benefit from observational research compared to this study.

This study has some limitations that affect the generalizability of the results beyond the NICU nurses’ community in Jeddah, Saudi Arabia. It is important to note that this study used self-reporting, which involves people providing responses based on their perceptions; hence, their responses may have been affected by the bias of social acceptance. Participants may not have been able to fully assess themselves through self-reports. In addition to the small sample size, responses were collected from only two NICUs in Jeddah. The findings may have differed if the survey had included a larger number of participants. Since the quiet-time implementation was limited to only one hour per day in our study, future research is required to assess the benefits of quiet time when a longer period is applied.

## Conclusions

This study found no statistically significant relationship between quiet-time implementation and the perceived stress, anxiety, or depression of the NICU nurses. However, participants reported that quiet time is a power booster and beneficial for babies in the NICU. Future research should recruit more NICU nurses from different health sectors in Saudi Arabia who apply the quiet time protocol. Future research should also identify the factors that lead NICU nurses to experience stress, anxiety, and depression in NICUs in Saudi Arabia.
